# A Comprehensive Overview of the Clinical, Electrophysiological, and Neuroimaging Features of BPAN: Insights From a New Case Series

**DOI:** 10.1002/acn3.70220

**Published:** 2025-10-15

**Authors:** Seda Susgun, Ozgu Kizek, Sibel Aylin Ugur Iseri, Ibrahim Kamaci, Ayse Deniz Elmali, Pinar Iscen, Berfin Gulkaya Guzel, Gul Yalcin Cakmakli, Bulent Elibol, Berril Donmez, Raif Cakmur, Pinar Topaloglu, Turkish NBIA Study Group, Abdullah Acar, Ahmet Acarer, Arzu Karabay, Asuman Ali, Ayla Barlas, Aysegul Gunduz, Banu Ozen Barut, Baris Baslo, Bilge Kocer, Bilgehan Mus, Birsen Karaman, Burcu Gokce Cokal, Cem Ismail Kucukali, Cenk Akbostanci, Ceyhun Sayman, Cagla Turan, Dilek Ince Gunal, Ebru Bilge Dirik, Ebru Erzurumluoglu, Elif Kocasoy Orhan, Enes Demiryurek, Emrah Yucesan, Ercan Kose, Erdem Tuzun, Esen Saka Topcuoglu, Esra Okuyucu, Fatma Betul Ozdilek, Feriha Ozer, Gencer Genc, Gozde Unal, Gulay Kenangil, Gullu Tarhan, Gunes Kiziltan, Halil Onder, Hamit Genc, Hasmet Hanagasi, Hatice Yuksel, Hulya Apaydin, Koray Kirimtay, Mehmet Guney Senol, Melisa Kilic, Meltem Demirkiran, Mert Karaca, Miray Erdem, Muhammet Bilgehan Mus, Murat Gultekin, Nalan Capan, Nazan Karagoz Sakalli, Nazli Basak, Nihan Hande Akcakaya, Ozan Ezer, Ozge Uygun, Ozge Yilmaz Kuspeci, Ozgur Oztop Cakmak, Pervin Iseri, Petek Ballar Kirmizibayrak, Pinar Elkoca, Recep Alp, Remzi Yigiter, Rezzak Yilmaz, Sadika Ozdemir, Selda Keskin, Selen Ilhan Alp, Selen Soylu, Serdar Ceylaner, Serhat Ozkan, Sevda Erer Ozbek, Sevgin Gundogan, Sevil Yasufli, Sezin Alpaydin Baslo, Sibel Ertan, Sultan Cagirici, Seyma Aykac, Vuslat Yilmaz, Yaprak Secil, Yasar Kutukcu, Yeliz Ciftci, Yesim Sucullu Karadag, Yildiz Değirmenci, Zeliha Matur, Nerses Bebek, Murat Emre, Zuhal Yapici

**Affiliations:** ^1^ Department of Genetics, Aziz Sancar Institute of Experimental Medicine Istanbul University Istanbul Turkiye; ^2^ Department of Medical Biology, Faculty of Medicine Bezmialem Vakif University Istanbul Turkiye; ^3^ Department of Neurology, Istanbul Faculty of Medicine Istanbul University Istanbul Turkiye; ^4^ Epilepsy Center (EPIMER), Istanbul Faculty of Medicine Istanbul University Istanbul Turkiye; ^5^ Department of Child and Adolescent Psychiatry, Istanbul Faculty of Medicine Istanbul University Istanbul Turkiye; ^6^ Department of Neurology, Faculty of Medicine Hacettepe University Ankara Turkiye; ^7^ Department of Neurology, Institute of Neurological Sciences and Psychiatry Hacettepe University School of Medicine Ankara Turkiye; ^8^ Department of Neurology, Faculty of Medicine Dokuz Eylul University Izmir Turkiye; ^9^ Division of Child Neurology, Department of Neurology, Istanbul Faculty of Medicine Istanbul University Istanbul Turkiye

**Keywords:** beta‐propeller protein‐associated neurodegeneration (BPAN), epilepsy, fluorodeoxyglucose positron emission tomography (FDG‐PET), magnetic resonance imaging (MRI), neurodegeneration with brain iron accumulation (NBIA), *WDR45*

## Abstract

**Background:**

Neurodegeneration with brain iron accumulation (NBIA) comprises a genetically and clinically heterogeneous group of rare neurological disorders characterized particularly by iron accumulation in the basal ganglia. To date, 15 genes have been associated with NBIA. Among them, *WDR45*, linked to beta‐propeller protein‐associated neurodegeneration (BPAN), represents the only X‐linked dominant subtype of NBIA. Herein, clinical, electrophysiological, and neuroimaging evaluations were used to broaden the understanding of BPAN in a newly reported case series.

**Methods:**

This study included 10 individuals with BPAN, categorized into three age groups. *WDR45* variant data retrieved from next‐generation sequencing or Sanger sequencing were reviewed and reassessed. Comprehensive clinical evaluations including magnetic resonance imaging (MRI), fluorodeoxyglucose positron emission tomography (FDG‐PET), and video electroencephalographic monitoring were conducted.

**Results:**

The clinical manifestations were highly heterogeneous, with cognitive impairment being a consistent finding among the patients, with variable severity. The associated *WDR45* variants are likely to exert loss‐of‐function effects. Electroencephalogram (EEG) abnormalities included age‐dependent background slowing and epileptiform discharges. MRI indicated a characteristic pattern, while two patients lacked these typical findings. FDG‐PET imaging demonstrated hypometabolism extending beyond cerebral structures, with predominant cerebellar and pontine involvement in pediatric patients and frontoparietal hypometabolism in adults.

**Conclusions:**

This study contributes further to our understanding of the heterogeneous clinical spectrum of BPAN. Genotype–phenotype correlation in BPAN remains unclear due to the absence of sufficiently large cohorts in the literature, including the present study. Nevertheless, even within this small sample, the phenotypic heterogeneity observed among individuals harboring the same genotype highlights the biological complexity of the disease. Neuroimaging findings may reflect progressive and widespread neurological involvement in an age‐dependent pattern, whereas EEG data suggest that epilepsy severity tends to decrease after adolescence.

## 1 Introduction

Neurodegeneration with brain iron accumulation (NBIA) comprises a clinically and genetically heterogeneous group of rare neurological disorders affecting both children and adults. To date, pathogenic variants in 15 genes have been implicated in NBIA, with a combined prevalence of 0.1–0.3 per 100,000 worldwide [1, 2, 3]. Basal ganglia iron accumulation, a hallmark of NBIA, occurs despite the lack of direct involvement of most associated genes in iron regulation, challenging its role in pathogenesis [4, 5]. Extrapyramidal, pyramidal, and cognitive impairments are typically the dominant clinical features in most NBIA subtypes, whereas seizures are generally rare [1, 6]. However, epilepsy is a recognized feature in several forms, including phospholipase A2‐associated neurodegeneration (PLAN), particularly its infantile neuroaxonal dystrophy (INAD) subtype, as well as in beta‐propeller protein‐associated neurodegeneration (BPAN), mitochondrial membrane protein‐associated neurodegeneration (MPAN), with BPAN exhibiting a particularly prominent epileptic phenotype, especially in childhood [1, 4, 6].

BPAN has increasingly been recognized as the most common NBIA subtype, seemingly predominating over PKAN in clinical frequency [1, 7, 8]. Clinically, BPAN shows a biphasic manifestation; it is characterized by developmental delay (DD), intellectual disability (ID), epilepsy, and autistic‐like features, which manifest in childhood as a first phase. In early adulthood, the disease enters into a second phase, marked by the onset and progression of extrapyramidal symptoms, including dystonia, parkinsonism, and dementia [7, 8, 9]. While the MRI findings, particularly the banded halo sign on T1‐weighted images, serve as a distinguishing radiological hallmark of BPAN, little is known about positron emission tomography (PET) findings in affected individuals. To the best of our knowledge, PET has been reported in only two studies to date, not allowing for definitive conclusions [10, 11]. Additionally, reports on electroencephalographic (EEG) findings in BPAN are limited and suggest a wide spectrum of abnormalities [12, 13, 14]. In some cases, EEG alterations may precede detectable changes on MRI. Although not pathognomonic, specific EEG patterns, particularly in females presenting with DD, ID, or epileptic seizures, may serve as early clues and warrant clinical attention [15].

BPAN is the only X‐linked dominant form of NBIA and is caused by pathogenic variants in the *WDR45* gene (MIM: *300526) [6]. *WDR45* encodes a WD‐repeat protein with an N‐terminal β‐propeller structure, although its precise function remains poorly understood. Its dysfunction disrupts autophagy, induces mitochondrial dysfunction and endoplasmic reticulum (ER) stress, and leads to iron dysregulation, emphasizing its role in multiple cellular pathways. Notably, pathogenic variants in *WDR45* have been associated with a spectrum of overlapping clinical presentations, including BPAN, ID, Rett‐like syndrome, developmental and epileptic encephalopathy, early‐onset epileptic encephalopathy, and West syndrome, highlighting its broad phenotypic spectrum [16]. Furthermore, a recent large‐scale study identified *WDR45* as one of the five most significant genes implicated across all epilepsy subtypes and their comorbidities [17].

In this study, we present a case series of 10 patients with *WDR45* variants, diagnosed and monitored between 2019 and 2024. In addition to comprehensive clinical assessments, our report includes detailed MRI and PET findings, epilepsy phenotypes supported by electrophysiological data, and genetic interpretations. Through this unique cohort, we aim to provide an in‐depth characterization of BPAN and to draw attention to specific clinical and diagnostic features that may have been underrecognized in the existing literature.

## 2 Patients and Methods

### 2.1 Clinical Assessments

This study has been approved by Istanbul University, Istanbul Faculty of Medicine, Clinical Ethics Committee (12/01/2023‐24). Written informed consents were obtained from all participants enrolled in the study according to the Helsinki Declaration. This study was conducted at the NBIA Research Center, a newly established unit within the Department of Neurology at Istanbul Faculty of Medicine, a national referral center for NBIA disorders in Turkiye. Patients, referred from external institutions or self‐referred, were hospitalized for an initial evaluation lasting approximately 2–3 weeks. A standardized diagnostic workup was applied, comprising detailed neurological examination, neuroimaging investigations, electrophysiological tests, targeted biochemical analyses, clinical scoring, genetic testing when indicated, and multidisciplinary consultations.

In the present study, a total of 10 patients diagnosed with BPAN were included. Comprehensive physical and neurological examinations were performed on all affected individuals and, where available, their family members. All patients underwent prolonged video‐EEG monitoring, lasting over 12 h, covering wakefulness, natural nocturnal sleep, and activation methods. EEG recordings were obtained using 20 scalp electrodes following the international 10–20 system, with additional electrodes for electrooculography (EOG) and chin electromyography (EMG) to facilitate sleep stage differentiation according to AASM v3.0 criteria [18]. For the activation procedures, repeated eye opening and eye closure were performed 10 times. Hyperventilation, lasting 5 min, was performed when feasible. Additionally, photic stimulation was applied using a broad frequency range, including unconventional lower frequencies (2, 4, 5, 8, 10, 12, 15, 18, 20, 25, and 30 Hz).

Cranial MRI was performed on all patients using a SIEMENS MAGNETOM 3.0T XQ Numaris scanner. Axial plane sequences included T1‐weighted imaging (T1WI) and susceptibility‐weighted imaging (SWI) with a slice thickness of 1 mm, as well as T2‐weighted imaging (T2WI) and diffusion‐weighted imaging (DWI) with a slice thickness of 3 mm. Coronal plane sequences consisted of T2WI with a slice thickness of 3 mm, while sagittal plane imaging included fluid‐attenuated inversion recovery (FLAIR) sequences with a slice thickness of 1 mm.

The PET scans using fluorine‐18 fluorodeoxyglucose (18F‐FDG) were performed as part of a standardized protocol established at the NBIA Research Center and were applied to all BPAN patients without contraindications, with the aim of exploring the potential of PET‐based imaging biomarkers in this relatively understudied field.

### 2.2 Genetic Analyses

Genetic testing was performed as part of clinical diagnosis or research studies, employing whole‐exome sequencing (WES), clinical‐exome sequencing (CES), or Sanger sequencing for the probands. The MANE (Matched Annotation from NCBI and EBI) Select transcripts have been used for all variant annotations (*WDR45*: ENST00000376372.9; NM_001029896.2).

## 3 Results

### 3.1 Clinical Assessments

Out of 106 NBIA cases followed at NBIA Research Center, 10 patients (9.4%) were diagnosed with BPAN. BPAN patients were categorized into three age groups: childhood (4.7 ± 1.8 years, *n* = 5), adolescence (13 years, *n* = 1), and adulthood (39.5 ± 2.6 years, *n* = 4). The cohort included six female and four male patients. The demographic, clinical, and electrophysiological characteristics of the patients are summarized in Table 1.

**TABLE 1:** Clinical features, electrophysiological and PET findings of the BPAN patients.

**Patient No**.	**1**	**2**	**3**	**4**	**5**	**6**	**7**	**8**	**9**	**10**
**Age at last evaluation**	20 mo	35 yo	7 yo	3.5 yo	5.5 yo	40 yo (deceased)	13 yo	5.5 yo	41 yo	42 yo
**Sex**	M	M	F	F	F	M	F	F	F	M
**Initial findings**	MDD, LDD	Mild motor delay, febrile seizure (2 times; between 3–6 yo), LDD	Mild motor delay, febrile seizure (3 times; between 2–3 yo), LDD	Unprovoked seizure, MDD, LDD	Febrile seizure (5 times; between 2–3 yo), MDD, LDD	LDD	MDD, LDD	MDD, LDD	Febrile seizure (7 times between 1 and 4 yo), LDD	Febrile seizure, MDD, LDD
**ID**	Yes	Yes	Yes	Yes	Yes	Yes	Yes	Yes	Yes	Yes
**Somatic neurological features**	Hypotonia Hyporeflexia No sitting No standing No walking Stereotypic movements	Bradymymia, Bradykinesia/ akinesia, Bilateral rigidity in all extremities, Dystonia in both feet with contracture (right>), Semiflexion posture of the wrists, elbows, hips, and knees, Postural instability, Standing with support (Extrapyramidal deterioration began at age 30)	Stereotypic movements	Hypotonia Hyporeflexia No sitting No standing No walking	Within normal limits	Bradymymia, Bradykinesia, Bilateral cogwheel rigidity, Rest and postural tremor (right>), Parkinsonian gait, Walking with support (Extrapyramidal deterioration began at age 26)	Bradymymia, Bradykinesia, Axial hypotonia, Semiflexion posture in upper extremities, and rigidity in upper extremities, Walking with support	Within normal limits	Bradymymia, Bradykinesia, Bilateral cogwheel rigidity, Rest and postural tremor, Bilateral hand dystonia, Walking without support (Extrapyramidal deterioration began at 35 age)	Bradymymia, Bradykinesia, Bilateral cogwheel rigidity, Bilateral distal hand dystonia and left foot dystonia, Action tremor, Stereotypic movements, Parkinsonian gait, Walking with support (Extrapyramidal deterioration began at age 28)
**Diagnosis of epilepsy**	Yes	No	Yes	Yes	No	NA	Yes	No	No	Yes
**Seizure onset age**	15 mo	—	5 yo	7 mo	—	—	1 yo	—	—	38 yo
**Type of seizure**	Behavioral arrest, starring	—	Generalized convulsion	Focal (7 mo), atonic (7 mo), epileptic tonic spasms (9 mo)	—	Behavioral arrest, starring was reported by family	Myoclonic, tonic	—	—	Generalized convulsion
**Antiseizure medications**	LEV	VPA	LEV, VPA, CLB, ACTH	LEV, PB, PHT, RUF, CBZ, TPM, ACTH, hydrocortisone	—	—	LEV, CBZ, PB, VGB, CLN, VPA, LTG, ketogenic diet	—	—	LEV
**Response to the treatment**	Partial response	—	Partial response	Intractable	—	Good	Intractable	—	—	Good
**Background activity**	Theta, delta	Theta, delta	Theta, delta	Delta	Delta	NA	Theta, delta	Theta	Alpha and theta	Theta, delta
**Fast activity**	Yes, 20–24 Hz	Yes, 20–23 Hz	Yes, 19–23 Hz	No	Yes, 20 Hz	NA	No	No	Yes, 16 Hz	No
**Epileptiform abnormality**	Frontal spike‐and‐waves, frontal, and occipital RDA	No	Temporal, centroparietal sharp waves, occipital RDA, SWAS (6 and 7 yo)	Multifocalspike‐and‐waves, tonic pattern, atonic seizures, burst‐suppression during sleep	No	NA	Frontal, central spike‐and‐waves, tonic pattern, atypical absence (Lennox Gastaut Syndrome)	No	No	No
**Asymmetry**	No	No	Yes	No	No	NA	No	No	No	No
**Brain PET**	NA	Hypometabolism in the bilateral inferior parietal (more prominent on the left), bilateral superior parietal (more prominent on the left), bilateral lateral frontal, and right lateral temporal regions	Hypometabolism in the cerebellum, and pons, bilateral mesial temporal, bilateral medial prefrontal (right>), right lateral prefrontal	Hypometabolism in the cerebellum, pons, bilateral mesial temporal, inferior parietal (left>), left medial and lateral prefrontal	NA	NA	Hypometabolism in the cerebellum, bilateral inferior parietal, bilateral lateral temporal	Hypometabolism in the cerebellum and pons, bilateral mesial temporal, left superior parietal, left medial and lateral prefrontal, bilateral occipital lateral, left posterior cingulate area	NA	Hypometabolism is severe and global, with a marked involvement of the bilateral frontoparietal regions in the cerebrum

*Note:* Recent anti‐seizure treatments of the patients are underlined.

Abbreviations: ACTH, adrenocorticotropic hormone treatment; CBZ, carbamazepine; CLB, clobazam; CLN, clonazepam; F, female; ID, intellectual disability; LDD, language developmental delay; LEV, levetiracetam; LTG, lamotrigine; M, male; MDD, motor developmental delay; mo, month‐old; NA, not available; PB, phenobarbital; PET, positron emission tomography; PHT, phenytoin; RDA, rhythmic delta activity; RUF, rufinamide; SWAS, spike wave action in sleep; VGB, vigabatrin; VPA, valproic acid; yo, year‐old.

All patients exhibited a history of delayed language development in childhood. Eight patients experienced delays or regression in motor milestones, while five had a history of self‐limited febrile seizures. In one patient (P4), unprovoked seizures together with developmental delay emerged as an early‐onset manifestation at the age of 7 months. At the time of genetic diagnosis, all patients exhibited clear clinical manifestations, at a minimum presenting with delayed language development, although two patients (P5 and P8) had normal neurological examinations. Among pediatric patients, three (P3, P5, and P8) were able to walk independently.

Longitudinal clinical histories of the four adult BPAN patients are outlined below (also see Table 1): Currently, four adult patients (three males: P2, P6, P10; one female: P9) are diagnosed with BPAN, ranging in age from 35 to 42 years. According to family reports, all patients exhibited delayed speech development in early childhood, either never achieving speech or losing all expressive language abilities by the age of five, despite initial progress. Three patients had a history of 2 to 7 febrile seizures between the ages of 1 and 6. Additionally, two patients demonstrated mild motor developmental delay, achieving independent walking around the age of 2 to 3 years. None of the patients experienced drug‐resistant epilepsy during childhood or adulthood. Only one male patient (P10) had a single generalized tonic–clonic seizure at the age of 38. Levetiracetam was started afterward, and he has been seizure‐free for the past 3 years.

All patients had previously been followed during childhood or early adulthood with presumptive diagnoses of cognitive impairment. Extrapyramidal symptoms developed between the ages of 26 and 35 in all four patients, progressing to moderate‐to‐severe parkinsonism within 1 to 2 years. Two male patients (P2 and P10) showed rapid disease progression after symptom onset at ages 28 and 30, respectively, becoming bedridden within 2 years. In contrast, one male patient (P6), whose symptoms began at age 26, remains ambulatory with minimal assistance at age 40, after 14 years of follow‐up. The female patient (P9), who developed symptoms at age 35, has been followed for 6 years and remains fully mobile and independent in daily activities at age 41.

### 3.2 Epilepsy and EEG Findings

When assessed for lifetime occurrence of epileptic seizures, 8 out of 10 patients were found to have experienced either provoked or unprovoked seizures (see Table 1). Five patients (P2, P3, P5, P9, and P10) experienced recurrent febrile seizures, two of whom subsequently developed epilepsy. A confirmed epilepsy diagnosis was present in five patients, including three children (P1, P3, and P4), one adolescent (P7), and one adult (P10). Seizure onset typically occurred in infancy or early childhood, except for P10, who developed epilepsy at 38 years of age. Seizure types varied significantly across patients, with some exhibiting a single seizure type, while others experienced multiple types. Levetiracetam provided limited and temporary seizure control in two patients, whereas two patients had drug‐resistant epilepsy. Seizure control was obtained, albeit transiently, in one patient treated with clobazam (P3), whereas valproate, clonazepam, rufinamide, and phenobarbital were discontinued due to insufficient efficacy (P4, P7). Notably, ACTH (adrenocorticotropic hormone) therapy achieved seizure control in two patients with epileptic encephalopathy: one with infantile spasms and the other with spike–wave activation during sleep (SWAS).

EEG findings were analyzed in nine patients; P6 could not undergo a standardized EEG examination. All EEGs revealed diffuse slowing of background activity, even after adjusting for age‐related background rhythm frequencies. Additionally, five patients exhibited diffuse fast background activity. In one patient (P1), this appeared as rhythmic discharges at 20–24 Hz, consistent with an epileptiform abnormality (Figure S1). Two patients (P2, P3) receiving benzodiazepine treatment displayed fast background rhythms. Among adult patients, EEG findings consisted of background slowing and fast activity, whereas pediatric patients exhibited more variable electrophysiological abnormalities. In addition to background slowing, focal epileptiform discharges, including spikes, polyspikes, and spike–wave patterns, were observed in the frontal, central, and temporal regions. Rhythmic delta activity was detected in the occipital and frontal regions of two patients (P1, P3).

Video‐EEG monitoring captured atypical absence, myoclonic, tonic, and atonic seizures in two patients (P4, P7). One of these patients (P4) exhibited a hypsarrhythmia pattern along with a burst‐suppression pattern during sleep (Figures S2 and S3). Additionally, one patient (P3) had a history of SWAS. She began crawling at 9 months, spoke her first words at 15 months, and started walking at 2 years of age. She experienced a febrile seizure at the age of 3 years, followed by the onset of epileptic seizures at the age of 4 years. Levetiracetam and valproic acid treatments were initiated. At the age of 5 years, EEG revealed a SWAS pattern. Following the initiation of ACTH therapy and the addition of clobazam, she has remained seizure‐free for the past 2 years. She is currently able to speak in two‐word phrases and can walk and run independently.

### 3.3 Neuroimaging Findings

Metabolic and functional brain alterations were investigated using FDG‐PET in 6 out of 10 patients. PET imaging revealed metabolic abnormalities extending beyond cerebral structures, with hypometabolism detected in the cerebellum and pons. Infratentorial involvement was more pronounced in pediatric patients, while frontoparietal hypometabolism was more prominent in adults (see Table 1).

MRI findings are summarized in Table 2. Characteristic MRI findings, such as the bilateral hyperintense halo sign in the SN, were present in eight patients. However, in P1 and P5, these typical MRI features were absent, while in P3, P4, and P8 the halo sign was only mildly noticeable. SN involvement appeared more prominent than GP, unlike the pattern typically observed in PKAN.

**TABLE 2:** MRI findings in the BPAN cohort.

Patient no.	1	2	3	4	5	6	7	8	9	10
Age at MRI	20 mo	35 yo	7 yo	3 yo	28 mo	37 yo	13 yo	5 yo	41 yo	42 yo
Basal ganglia on T1‐WI	No halo sign in the SN	Bilatreral hyperintense halo sign in the SN	Bilatreral hyperintense halo sign in the SN (mildly)	Bilatreral hyperintense halo sign in the SN (mildly)	No halo sign in the SN	Bilatreral hyperintense halo sign in the SN	Bilatreral hyperintense halo sign in the SN	Bilatreral hyperintense halo sign in the SN (mildly)	Bilatreral hyperintense halo sign in the SN	Bilatreral hyperintense halo sign in the SN and GP
Basal ganglia on T2‐WI	No hypointensity	Bilateral hypointensity in the GP and the SN	Bilateral hypointensity in the SN	Bilateral hypointensity in the SN (mildly)	No hypointensity	Bilateral hypointensity in the GP and the SN	Bilateral hypointensity in the GP and the SN	Bilateral hypointensity in the SN	Bilateral hypointensity in the GP and the SN	Bilateral hypointensity in the GP and the SN
Basal ganglia on SWI	No hypointensity	Bilateral hypointensity in the GP and the SN	Bilateral hypointensity in the SN	Bilateral hypointensity in the SN	No hypointensity	Bilateral hypointensity in the GP and the SN	Bilateral hypointensity in the GP and the SN	Bilateral hypointensity in the SN	Bilateral hypointensity in the GP and the SN	Bilateral hypointensity in the GP and the SN
Corpus Callosum	Diffuse thinning	N	Diffuse thinning	Diffuse thinning	Diffuse thinning	Diffuse thinning	Diffuse thinning	Diffuse thinning	N	Diffuse thinning
Dentate nucleus on T2‐WI	Bilateral symmetric hyperintensity and swelling	N	N	Bilateral symmetric hyperintensity	N	N	N	Bilateral symmetric hyperintensity (very mild)	N	Bilateral symmetric hyperintensity (mild)
White matter	Diffuse myelin reduction	N	N	N	N	N	N	Bilateral nonspesific hiperintens pale areas	N	N
Cortical/subcortical atrophy	Mild	Moderate–severe	Mild	Mild	No	Severe (mostly cortical)	Mild	Mild cerebellar	Severe (mostly cortical)	Severe (mostly cortical)

Abbreviations: GP, globus pallidus; mo, month‐old; MRI, magnetic resonance imaging; N, normal; SN, substantia nigra; SWI, susceptibility‐weighted imaging; T1‐WI, T1‐weighted imaging; T2‐WI, T2‐weighted imaging; yo, year‐old.

### 3.4 Genetic Analyses

Nine different *WDR45* variants were identified in 10 patients (Table 3). Among these, (i) six variants result in a premature stop codon at least 55 bp upstream of the last exon‐exon junction, (ii) one variant led to the loss of the initiation codon, and (iii) two variants affect splice sites. None of these variants are reported in public databases such as gnomAD (v4.1.0) and 1000 Genomes. Eight variants are classified as high‐impact and pathogenic, while one variant (c.235+5G>A) is considered a variant of uncertain significance (VUS) with low‐impact, according to both Ensembl Variant Effect Predictor (VEP) tool and GeneBe Variant Interpretation tool [19, 20]. The variants were hemizygous in males and heterozygous in females, as expected. Dual parental data was available only for P10, allowing the assumption of its de novo status. P6 and P10 were suggestive of mosaicism for the variant [21, 22].

**TABLE 3:** Identified *WDR45* variants in the patients with BPAN. Variants were annotated through the Ensembl VEP, MANE Select transcripts are presented (last accession date: 02/11/2025).

Patient no.	Location (hg38)	HGVSc (ENST00000376372.9; NM_001029896.2)	HGVSp (ENSP00000365551.3)	Consequence	VEP impact	Exon	dbSNP	ClinVar	References
1	X:49078094‐49078094	c.2T>C	p.Met1?	Start lost	High	2/11	rs1569523565	P (2)	This study
2	X:49078077‐49078077	c.19C>T	p.Arg7Ter	Stop gained	High	2/11	rs886041382	P (8)	This study
3	X:49077638‐49077638	c.235+5G>A	—	Splice donor 5th base variant, intron variant	Low	Int 4/10	rs2065045234	P (1); VUS (1)	This study
4–5	X:49076469‐49076469	c.397C>T	p.Arg133Ter	Stop gained	High	6/11	rs797046101	P (9); LP (1)	This study
6	X:49075940‐49075940	c.442dup	p.Cys148LeufsTer2	Frameshift variant	High	7/11	—	NA	Susgun et al. [22]
7	X:49075862‐49075865	c.516+1_516+3del	—	Splice donor variant, splice donor region variant, intron variant	High	Int 7/10	—	P (2); LP (1)	This study
8	X:49075573‐49075573	c.697C>T	p.Arg233Ter	Stop gained	High	8/11	rs387907329	P (12)	This study
9	X:49075432‐49075432	c.759C>A	p.Cys253Ter	Stop gained	High	9/11	—	NA	This study
10	X:49075239‐49075239	c.870C>G	p.Tyr290Ter	Stop gained	High	10/11	rs782557596	P (3)	Akcakaya et al. [21]

Abbreviations: Int, intron; LP, likely pathogenic; NA, not available; P, pathogenic; VUS, variant of uncertain significance.

For the splicing variants, SpliceAI analysis predicts that the c.516+1_516+3del results in significant donor loss (score: 0.94), whereas c.235+5G>A has a minor predicted effect on splicing (acceptor gain score: 0.07) (via Ensembl VEP, last accession date: 02/11/2025). Consistently, Human Splicing Finder (HSF) indicates that c.516+1_516+3del disrupts the wild‐type donor site, leading to impaired splicing, while c.235+5G>A shows no significant effect (HSF Pro Mutations Analysis Version 2.05 (2022‐09‐12), last accession date: 02/11/2025). However, the MaxEntScan suggests that the c.235+5G>A may alter splicing by creating a cryptic splice site or causing exon skipping (score: 8.76, difference: 2.10) (via Ensembl VEP, last accession date: 02/11/2025). Thus, the pathogenicity of the c.235+5G>A requires further confirmation through additional functional studies. Yet, based on clinical findings consistent with the disease, the patient (P3) received a clinical diagnosis of BPAN; therefore, we considered including the patient in this cohort.

## 4 Discussion

Among the NBIA spectrum, BPAN is considered one of the more commonly observed subtypes; however, significant gaps persist in our understanding of its phenotypic spectrum and underlying pathophysiology. Despite increasing recognition, its rarity in the general population and complexity of its clinical course frequently leads to diagnostic delays.

In particular, early recognition of BPAN remains challenging, particularly in female infants presenting with infantile spasms, where the diagnosis is often overlooked. Several diagnostic clues have been identified, including language and developmental delay, epilepsy, characteristic EEG biomarkers, and MRI features such as corpus callosum thinning and generalized atrophy. These clinical and radiological findings collectively suggest a widespread impact of *WDR45*‐related autophagy dysfunction, potentially contributing to both epileptogenesis and neurodegeneration in BPAN. To provide a more detailed clinical understanding, we conducted a comprehensive clinical, electrophysiological, neuroimaging, and genetic analysis of 10 BPAN patients, highlighting the age‐dependent phenotypic spectrum, epileptic manifestations, and the impact of *WDR45* variants on disease progression.

### 4.1 Epilepsy, Electrophysiological and Neuroimaging Findings

Epilepsy is a well‐recognized comorbidity in BPAN, with a reported prevalence of 67.7%, according to a recent review [23]. This highlights the significant burden of seizures in BPAN and underscores the need for a deeper understanding of its epileptic manifestations. Seizure severity in BPAN spans a broad spectrum, ranging from mild febrile seizures requiring no treatment to drug‐resistant epilepsy with multiple seizure types, including generalized, focal, and syndromic presentations such as infantile epileptic spasms syndrome and Lennox–Gastaut syndrome [14, 24, 25, 26]. As anticipated, half of our patients had febrile seizures in childhood; while some remained seizure‐free thereafter, others developed a range of seizure types with varying treatment responses and heterogeneous EEG findings. Although there is no consensus in the literature regarding a standard anti‐seizure treatment [8, 27], ACTH and prednisolone have been reported to be effective in controlling seizures in cases consistent with epileptic spasms [28, 29]. In the seven‐patient cohort reported by Carvill et al., valproate was ineffective or seizure‐aggravating in most cases, with only one patient showing partial benefit [12]. ACTH and prednisolone provided some improvement in infantile spasms and hypsarrhythmia, while clobazam was effective in two patients but worsened seizures in two others. Lamotrigine and topiramate showed no efficacy. Stige et al. described a single case with sustained seizure control on valproate, whereas Chen et al. reported partial or absent response [23, 30]. In our adult patients, epilepsy was not a predominant clinical manifestation, and children with only fever‐provoked seizures remained seizure‐free with or without medication. Consistent with the heterogeneous responses reported in the literature, our experience with BPAN patients with intractable epilepsy also indicates that none of these treatments achieved complete remission; their effects were limited to partial and often transient benefit, as seen with clobazam, ACTH, valproate, the valproate–lamotrigine combination, or ketogenic diet. In these patients with intractable epilepsy, antiseizure medications appeared initially effective but rapidly lost efficacy within weeks or months, and they consistently required polytherapy, without achieving sustained seizure freedom. Overall, current evidence, including our data, does not support any specific drug as consistently effective in BPAN.

Background slowing and low‐voltage activity are hallmark EEG findings in BPAN [12, 30]. In our cohort, all patients consistently exhibited diffuse background slowing. Notably, diffuse superimposed beta activity, previously suggested as a potential early biomarker, was also observed [13, 27]. Although this pattern is typically seen during drowsiness in children under 3 years of age, we identified it in two adult patients as well, suggesting that it may not be confined to the pediatric population. Importantly, this beta activity was present in both benzodiazepine‐treated and untreated individuals, implying that it may represent an intrinsic neurophysiological feature of BPAN rather than a pharmacological effect. This observation could therefore aid in earlier recognition and diagnosis, particularly when considered alongside other clinical and electrophysiological findings.

Epileptiform discharges, both focal and generalized, are frequently reported in BPAN [24]. Documented patterns include bilateral frontal spikes, occipital spikes, multifocal spikes, and 3 Hz spike–wave complexes, along with sleep‐related epileptiform spikes and SWAS and hypsarrhythmia in some cases [12, 14, 26, 28, 29, 30, 31, 32]. Prominent epileptiform abnormalities were observed in four pediatric patients; in one adolescent patient, the EEG findings were consistent with Lennox–Gastaut syndrome. In contrast, the absence of epileptiform discharges in our three adult patients supports prior observations of reduced seizure burden in adulthood; however, this electrophysiological finding does not conclusively indicate clinical seizure freedom [8, 23]. Nonetheless, since we lack previous EEG recordings of these adult patients, it is almost impossible to draw robust conclusions regarding the long‐term evolution of each individual patient's electrophysiological findings.

PET studies in BPAN remain limited in the NBIA literature, with only one case report describing frontal and left parietal hypometabolism in an adult patient [10]. Another study involving pediatric NBIA cases reported cerebellar and occipital hypometabolism [11]. Consistent with these two reports, PET scans in our cohort revealed widespread metabolic abnormalities. Infratentorial involvement, particularly of the cerebellum, appeared more pronounced in children, whereas frontoparietal hypometabolism was more consistently observed in adults. This distribution may reflect age‐related disease evolution and appears to be in line with the later presentation of extrapyramidal symptoms and cognitive decline. Additionally, mesial temporal involvement in three out of four pediatric patients may suggest a possible association between these structures and the greater seizure burden observed during childhood. Due to the scarce number of patients and the cross‐sectional nature of PET imaging, findings lack the ability to reflect the ontogenetic process precisely. However, even in the face of these limitations, we believe these PET findings are coherent with what we already know about the biphasic nature of disease manifestation. Therefore, we believe a longitudinal PET study in such patients could provide valuable information regarding the ontogenesis.

In pediatric cohorts, our findings contrast with those of Papandreou et al., whose large BPAN series (ages 0.8–14 years) did not demonstrate the T1 halo sign [7]. In our pediatric subgroup, however, the halo sign was present in several cases (Table 2), suggesting that this feature can occasionally be observed earlier in childhood than previously appreciated. Such differences may partly reflect methodological factors, as all MRIs in our series were acquired with a standardized 3T protocol; contributions from individual patient characteristics and genetic variant heterogeneity cannot be excluded. While earlier reports indicated that iron deposition becomes more readily detectable on SWI after 5 years of age [7], our results demonstrate that, with optimized protocols, the T1 halo and SWI hypointensities may already be evident from as early as 3 years. These findings also included cortical–subcortical atrophy (9/10 patients), corpus callosum thinning (8/10 patients), and dentate nucleus hyperintensity on T2‐weighted sequences in younger cases, features that have also been reported in some cases by other groups [7, 33]. When interpreted alongside clinical, EEG, and epilepsy‐related data, these imaging features may serve as critical biomarkers for early BPAN recognition, particularly in situations where hallmark signs such as the halo are inconspicuous.

### 4.2 Genetic Findings: What Matters for the Clinician?

We investigated whether specific clinical patterns could be associated with variant type, patient sex, or age. Among female patients with LoF variants in childhood (P4, P5, and P8), only P4 had epilepsy, whereas P3 (childhood) and P7 (adolescence), both carrying splicing variants, exhibited epilepsy. In males, P1 (childhood), with a start‐lost variant, had epilepsy, while among adult males with LoF variants (P2, P6, and P10), only P10 had late‐onset epilepsy. Phenotypic variability was evident even among patients carrying the same variant. P7 and a previously reported case [34] both carried the c.516+1_516+3del variant; however, while P7 experienced almost daily seizures, the reported case exhibited non‐progressive psychomotor retardation and cognitive dysfunction in childhood, later developing severe dystonia and gait disturbance after the age of 30. Similarly, P8 and a previously described patient [35] both harbored the p.Arg233Ter variant. Unlike P8, who displayed only mild cognitive and language impairments, the reported case presented with severe developmental delay, seizures, and atelencephaly. Interestingly, even in patients with identical variants, sex, and age parameters, phenotypic differences were observed. P4 and P5, both carrying the p.Arg133Ter variant, differed significantly: P4 exhibited epilepsy and a severe neurological phenotype, whereas P5 did not develop epilepsy or major neurological deficits. Although our cohort size limits definitive genotype–phenotype correlations, both our findings and prior reports suggest that such associations in BPAN are difficult to establish. This complexity may reflect the influence of additional genetic or epigenetic modifiers, consistent with emerging evidence implicating *WDR45* in epilepsy‐related pathways.

Given the growing recognition of *WDR45* in epilepsy genetics, elucidating its functional roles at the cellular level has become essential. A recent study involving the largest whole‐exome sequencing cohort in epilepsy identified ultra‐rare protein‐truncating variants in *WDR45* as one of five genes significantly associated with distinct epilepsy subtypes and comorbidities [17]. Notably, the odds ratio for *WDR45* approached that of *SCN1A*, a well‐established epilepsy gene, highlighting its emerging importance in epilepsy research in addition to its known role in neurodevelopmental disorders [17].

The pathogenic relevance of *WDR45* is reinforced by its role in autophagy and ER stress regulation, processes essential for neuronal homeostasis. Loss‐of‐function variants, as seen in BPAN, likely impair these pathways, disrupting neurodevelopment from early embryogenesis. This disruption extends beyond basal ganglia involvement, as structural and metabolic imaging reveals cortical and subcortical abnormalities, including FDG‐PET hypometabolism and atypical MRI findings. These observations suggest that *WDR45*‐related pathology affects widespread neural networks, potentially underlining the broad clinical spectrum encompassing epilepsy, cognitive impairment, and motor dysfunction.

These pathogenic mechanisms are further shaped by the gene's X‐linked inheritance pattern and variant‐specific molecular consequences. As an X‐linked disorder, BPAN predominantly affects females, with male cases being relatively rare and occasionally associated with mosaic *WDR45* variants [15, 21, 22, 36, 37]. Most *WDR45* variants (~88.5%) lead to LoF consequences, supporting a disease mechanism driven by absent and/or reduced protein function [16]. In our cohort, 80% of the variants can be reliably considered as LoF, including seven premature stop codons and one initiation loss variant. The remaining two patients carried splice site variants, one of which (c.516+1_516+3del) likely disrupts splicing, while the effect of c.235+5G>A remains uncertain. Functional studies suggest that +5 substitutions can impair splicing through U1 and U6 snRNAs [38], a mechanism also observed in patient fibroblasts [39, 40]. Accordingly, these two splice site variants may also lead to LoF by altering mRNA architecture. Such variant‐specific effects, combined with the gene's X‐linked nature, may partially explain the broad clinical spectrum observed in BPAN. As is known, X‐linked disorders generally manifest more severely in males, while females exhibit variable expression due to X‐chromosome inactivation (XCI) [41]. Stochastic events in XX females, including skewed XCI, somatic selection, and the timing and distribution of post‐zygotic mutations, may contribute to phenotypic variability [42]. Additionally, modifying genes and genetic background may further influence BPAN expressivity. A similar repertoire of stochastic events, except for XCI, may also apply to XY males. Furthermore, it should also be kept in mind that some patients may have unidentified X‐chromosome aneuploidies, which could further complicate phenotypic variability among BPAN patients.

## 5 Conclusion

In this study, we provide a comprehensive characterization of clinical, electrophysiological, neuroimaging, and genetic features of 10 patients with BPAN, thereby expanding the understanding of its phenotypic spectrum. Our findings highlight the wide range of epileptic manifestations and indicate that EEG alterations may precede MRI changes, whereas characteristic MRI features such as the T1 halo sign and SWI hypointensities can occasionally be detected earlier in childhood than previously appreciated. PET imaging revealed age‐dependent metabolic patterns, with infratentorial involvement in children and frontoparietal predominance in adults, suggesting a maturational shift and widespread network involvement. The variability observed even among patients carrying the same *WDR45* variant points to additional genetic, epigenetic, and stochastic influences, which may be particularly relevant in females. Taken together, these observations emphasize the importance of early recognition through integrated clinical, electrophysiological, and imaging assessments, and underline the need for longitudinal multicenter studies to better delineate disease trajectories and inform therapeutic strategies.

## Author Contributions

Conceptualization: Z.Y., S.S., S.A.U.I.; methodology: Z.Y.; formal analysis: S.S., S.A.U.I., O.K., A.D.E., I.K.; data curation: Z.Y., I.K., S.S., S.A.U.I., O.K., A.D.E.; Investigation: Z.Y., S.A.U.I., S.S., O.K., A.D.E., I.K., P.I., B.G.G., G.Y.C., B.E., B.D., R.C., P.T., N.B., M.E.; writing, draft preparation: S.S., S.A.U.I., O.K., A.D.E.; writing – review and editing: Z.Y., S.S., S.A.U.I., O.K., A.D.E.; supervision: Z.Y., S.S., S.A.U.I.

## Ethics Statement

This study has been approved by Istanbul University, Istanbul Faculty of Medicine, Clinical Ethics Committee (12/01/2023‐24). Written informed consents were obtained from all participants enrolled in the study according to the Helsinki Declaration. We confirm that we have read the Journal's position on issues involved in ethical publication and affirm that this report is consistent with those guidelines.

## Conflicts of Interest

The authors declare no conflicts of interest. ChatGPT (version 2025‐02‐06) was used solely for language editing and readability enhancement.

## Supporting information


**Figure S1:** Diffuse 20–24 Hz high amplitude fast activity. Longitudinal bipolar montage, paper speed 15 mm/s, sensitivity 100 μV/cm (P1).
**Figure S2:** Tonic pattern. Longitudinal bipolar montage, paper speed 15 mm/s, sensitivity 100 μV/cm (P4).
**Figure S3:** Burst‐suppression pattern during sleep. Longitudinal bipolar montage, paper speed 15 mm/s, sensitivity 100 μV/cm (P4).

## Data Availability

The data that support the findings of this study are available from the corresponding author upon reasonable request.

## Appendix A Turkish NBIA Study Group

Abdullah Acar (Department of Neurology, Dicle University, Faculty of Medicine, Diyarbakir, Turkiye), Ahmet Acarer (Department of Neurology, Faculty of Medicine, Ege University, Izmir, Turkiye), Arzu Karabay (Department of Molecular Biology and Genetics, Istanbul Technical University, Istanbul, Turkiye), Asuman Ali (Department of Neurology, Bursa Yuksek Ihtisas Training and Research Hospital, Bursa, Turkiye), Ayla Barlas (Department of Neurology, Istanbul Faculty of Medicine, Istanbul University, Istanbul, Turkiye), Aysegul Gunduz (Department of Neurology, Cerrahpasa Medical Faculty, Istanbul University‐Cerrahpasa, Istanbul, Turkiye), Banu Ozen Barut (Department of Neurology, University of Health Sciences Istanbul Kartal Dr. Lutfi Kirdar City Hospital, University of Health Sciences, Istanbul, Turkiye), Baris Baslo (Department of Neurology, Istanbul Faculty of Medicine, Istanbul University, Istanbul, Turkiye), Bilge Kocer (Department of Neurology, Ankara Etlik City Hospital, University of Health Sciences, Ankara, Turkiye), Bilgehan Mus (Department of Neurology, Istanbul Faculty of Medicine, Istanbul University, Istanbul, Turkiye), Birsen Karaman (Department of Medical Genetics, Istanbul Faculty of Medicine, Istanbul University, Istanbul, Turkiye), Burcu Gokce Cokal (Department of Neurology, Ankara Training and Research Hospital, University of Health Sciences, Ankara, Turkiye), Cem Ismail Kucukali (Department of Neuroscience, Aziz Sancar Institute of Experimental Medicine, Istanbul University, Istanbul, Turkiye), Cenk Akbostanci (Department of Neurology, School of Medicine, Ankara University, Ankara, Turkiye), Ceyhun Sayman (Department of Neurology and Neuroscience, Alanya Alaaddin Keykubat University, Antalya, Turkiye), Cagla Turan (Graduate School of Health Sciences, Istanbul University, Istanbul, Turkiye), Dilek Ince Gunal (Department of Neurology, Pendik Research and Training Hospital, Marmara University, Istanbul, Turkiye), Ebru Bilge Dirik (Department of Neurology, Ankara Bilkent City Hospital, Ankara, Turkiye), Ebru Erzurumluoglu (Department of Medical Genetics, Faculty of Medicine, Eskisehir Osmangazi University, Eskisehir, Turkiye), Elif Kocasoy Orhan (Department of Neurology, Istanbul Faculty of Medicine, Istanbul University, Istanbul, Turkiye), Enes Demiryurek (Department of Neurology, Faculty of Medicine, Istanbul Aydin University, Istanbul, Turkiye), Emrah Yucesan (Department of Neurogenetics, Institute of Neurological Sciences, Istanbul University‐Cerrahpasa, Istanbul, Turkiye), Ercan Kose (Department of Neurology, Gulhane Training and Research Hospital, Ankara, Turkiye), Erdem Tuzun (Department of Neuroscience, Aziz Sancar Institute of Experimental Medicine, Istanbul University, Istanbul, Turkiye), Esen Saka Topcuoglu (Department of Neurology, Faculty of Medicine, Hacettepe University, Ankara, Turkiye), Esra Okuyucu (Department of Neurology, Atilim University Faculty of Medicine, Ankara, Turkiye), Fatma Betul Ozdilek (Department of Neurology, Faculty of Medicine, Istanbul Medeniyet University, Istanbul, Turkiye), Feriha Ozer (Neurology Clinic, Hospital of Koç University, Istanbul, Turkiye), Gencer Genc (Department of Neurology, Sisli Hamidiye Etfal Training and Research Hospital, University of Health Sciences, Istanbul, Turkiye), Gozde Unal (Department of Neurology, Kanuni Sultan Suleyman Training and Research Hospital, Istanbul, Türkiye), Gulay Kenangil (Department of Neurology, Medipol Acibadem Hospital, Istanbul Medipol University, Istanbul, Turkiye), Gullu Tarhan (Bitlis State Hospital, Department of Neurology, Bitlis, Turkiye), Gunes Kiziltan (Department of Neurology, Cerrahpasa Medical Faculty, Istanbul University‐Cerrahpasa, Istanbul, Turkiye), Halil Onder (Department of Neurology, Ankara Etlik City Hospital, University of Health Sciences, Ankara, Turkiye), Hamit Genc (Neurology Clinic, Gaziantep City Hospital, Health Application and Research Center, Gaziantep, Turkiye), Hasmet Hanagasi (Department of Neurology, Istanbul Faculty of Medicine, Istanbul University, Istanbul, Turkiye), Hatice Yuksel (Department of Neurology, Ankara Bilkent City Hospital, Ankara, Turkiye), Hulya Apaydin (Department of Neurology, Cerrahpasa Medical Faculty, Istanbul University‐Cerrahpasa, Istanbul, Turkiye), Koray Kirimtay (Department of Molecular Biology and Genetics, Istanbul Technical University, Istanbul, Turkiye), Mehmet Guney Senol (Department of Neurology, Emsey Hospital, İstanbul, Turkiye), Melisa Kilic (Graduate School of Health Sciences, Istanbul University, Istanbul, Turkiye; Department of Genetics, Aziz Sancar Institute of Experimental Medicine, Istanbul University, Istanbul, Turkiye), Meltem Demirkiran (Department of Neurology, School of Medicine, Cukurova University, Adana, Turkiye), Mert Karaca (Department of Neurology, Istanbul Faculty of Medicine, Istanbul University, Istanbul, Turkiye), Miray Erdem (Department of Neurology, Adana City Training and Research Hospital, University of Health Sciences, Adana, Turkiye), Muhammet Bilgehan Mus (Department of Neurology, Istanbul Faculty of Medicine, Istanbul University, Istanbul, Turkiye), Murat Gultekin (Department of Neurology, Faculty of Medicine, Erciyes University, Kayseri, Turkiye), Nalan Capan (Department of Physical Medicine and Rehabilitation, Istanbul Faculty of Medicine, Istanbul University, Istanbul, Turkiye), Nazan Karagoz Sakalli (Department of Neurology, Bakirkoy Training and Research Hospital for Neurologic and Psychiatric Diseases, University of Health Sciences, Istanbul, Turkiye), Nazli Basak (Suna and Inan Kirac Foundation Neurodegeneration Research Laboratory NDAL‐ KUTTAM, School of Medicine, Koc University, Istanbul, Turkiye), Nihan Hande Akcakaya (Council of Forensic Medicine, Republic of Turkiye Ministry of Justice, Istanbul, Turkiye), Ozan Ezer (Department of Neurology, Istanbul Faculty of Medicine, Istanbul University, Istanbul, Turkiye), Ozge Uygun (Department of Neurology, Cerrahpasa Medical Faculty, Istanbul University‐Cerrahpasa, Istanbul, Turkiye), Ozge Yilmaz Kuspeci (Department of Neurology, IEU School of Medicine, Izmir, Turkiye), Ozgur Oztop Cakmak (Department of Neurology, School of Medicine, Koç University, İstanbul, Turkiye), Pervin Iseri (Department of Neurology, School of Medicine, Kocaeli University, Kocaeli, Turkiye), Petek Ballar Kirmizibayrak (Department of Biochemistry, Faculty of Pharmacy, Ege University, Izmir, Turkiye), Pinar Elkoca (Department of Neurology, Istanbul Faculty of Medicine, Istanbul University, Istanbul, Turkiye), Recep Alp (Private Neurology Clinic, Tekirdag, Turkiye), Remzi Yigiter (Department of Neurology, Medical Point Hospital, Gaziantep, Turkiye), Rezzak Yilmaz (Department of Neurology, School of Medicine, Ankara University, Ankara, Turkiye), Sadika Ozdemir (Department of Neurology, Bakirkoy Training and Research Hospital for Neurologic and Psychiatric Diseases, University of Health Sciences, Istanbul, Turkiye), Selda Keskin (Department of Neurology, Ankara Training and Research Hospital, University of Health Sciences, Ankara, Turkiye), Selen Ilhan Alp (Department of Neurology, Vocational School of Health Services, Namik Kemal University, Tekirdag, Turkiye), Selen Soylu (Department of Neuroscience, Aziz Sancar Institute of Experimental Medicine, Istanbul University, Istanbul, Turkiye), Serdar Ceylaner (Intergen Genetic and Rare Diseases Diagnosis and Research Center, Ankara, Turkiye), Serhat Ozkan (Department of Neurology, Faculty of Medicine, Eskişehir Osmangazi University, Eskişehir, Turkiye), Sevda Erer Ozbek (Department of Neurology, Faculty of Medicine, Uludag University, Bursa, Turkiye), Sevgin Gundogan (Department of Neurology, Ataturk Training and Research Hospital, Izmir Katip Celebi University, Izmir, Turkiye), Sevil Yasufli (Department of Neurology, Istanbul Faculty of Medicine, Istanbul University, Istanbul, Turkiye), Sezin Alpaydin Baslo (Department of Neurology, Bakirkoy Training and Research Hospital for Neurologic and Psychiatric Diseases, University of Health Sciences, Istanbul, Turkiye), Sibel Ertan (Department of Neurology, School of Medicine, Koç University, İstanbul, Turkiye), Sultan Cagirici (Department of Neurology, Servergazi State Hospital, Denizli, Turkiye), Seyma Aykac (Department of Neurology, Faculty of Medicine, Ege University, Izmir, Turkiye), Vuslat Yilmaz (Department of Neuroscience, Aziz Sancar Institute of Experimental Medicine, Istanbul University, Istanbul, Turkiye), Yaprak Secil (Department of Neurology, Ataturk Training and Research Hospital, Izmir Katip Celebi University, Izmir, Turkiye), Yasar Kutukcu (Department of Neurology, Anadolu Medical Center, Kocaeli, Turkiye), Yeliz Ciftci (Department of Neurology, Cigli Training and Research Hospital, Bakircay University, Izmir, Turkiye), Yesim Sucullu Karadag (Department of Neurology, Ankara Bilkent City Hospital, Ankara, Turkiye), Yildiz Değirmenci (Department of Neurology, Faculty of Medicine, Istanbul Health and Technology University, Istanbul, Turkiye), Zeliha Matur (Department of Neurology, Faculty of Medicine, Bezmialem Vakif University, Istanbul, Turkiye). Sibel Aylin Ugur Iseri (Department of Genetics, Aziz Sancar Institute of Experimental Medicine, Istanbul University, Istanbul, Turkiye) Ibrahim Kamaci (Department of Neurology, Istanbul Faculty of Medicine, Istanbul University, Istanbul, Turkiye) Pinar Iscen (Department of Neurology, Istanbul Faculty of Medicine, Istanbul University, Istanbul, Turkiye) Berfin Gulkaya Guzel (Department of Child and Adolescent Psychiatry, Istanbul Faculty of Medicine, Istanbul University, Istanbul, Turkiye) Gul Yalcin Cakmakli (Department of Neurology, Faculty of Medicine, Hacettepe University, Ankara, Turkiye.) Bulent Elibol (Department of Neurology, Institute of Neurological Sciences and Psychiatry, Hacettepe University School of Medicine, Ankara, Turkiye) Berril Donmez (Department of Neurology, Faculty of Medicine, Dokuz Eylul University, Izmir, Turkiye.) Raif Cakmur (Department of Neurology, Faculty of Medicine, Dokuz Eylul University, Izmir, Turkiye.) Murat Emre ( Department of Neurology, Istanbul Faculty of Medicine, Istanbul University, Istanbul, Turkiye.) Zuhal Yapici (Department of Neurology, Istanbul Faculty of Medicine, Istanbul University, Istanbul, Turkiye.)
