# Supplementary material for: A Comprehensive Overview of the Clinical, Electrophysiological, and Neuroimaging Features of BPAN: Insights From a New Case Series
Source: Ann Clin Transl Neurol. 2025 Oct 15;13(3):453–65. doi: 10.1002/acn3.70220 (PMC12968449; doi:10.1002/acn3.70220)
Supplement: Supplementary file 1 — Figure S1: Diffuse 20–24 Hz high amplitude fast activity. Longitudinal bipolar montage, paper speed 15 mm/s, sensitivity 100 μV/cm (P1). Figure S2: Tonic pattern. Longitudinal bipolar montage, paper speed 15 mm/s, sensitivity 100 μV/cm (P4). Figure S3: Burst‐suppression pattern during sleep. Longitudinal bipolar montage, paper speed 15 mm/s, sensitivity 100 μV/cm (P4). [file ACN3-13-453-s001.docx]

**Supplementary Material**


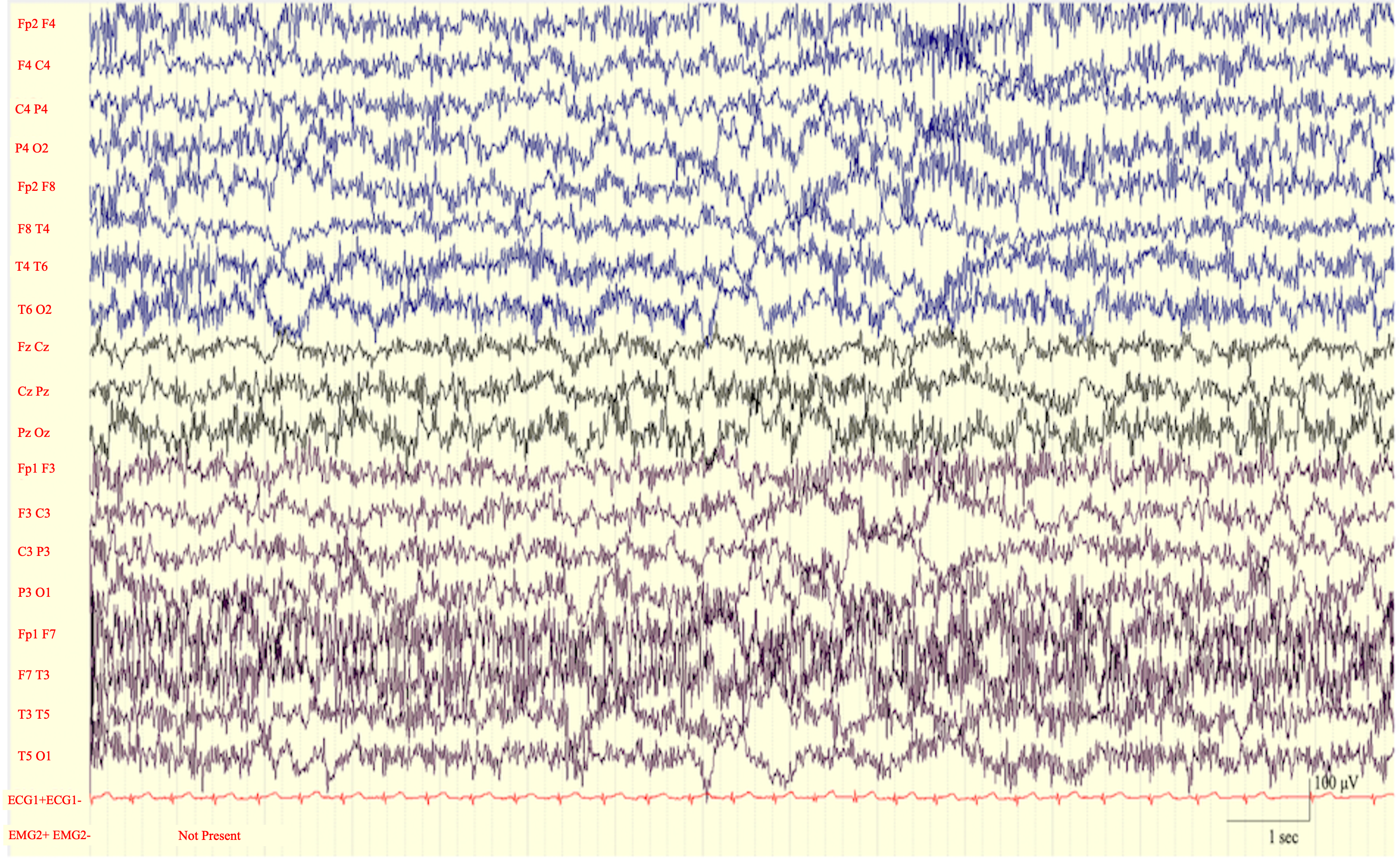
**Supplementary Figure 1.** Diffuse 20-24 Hz high amplitude fast activity. Longitudinal bipolar montage, paper speed 15 mm/s, sensitivity 100 µV/cm (P1).


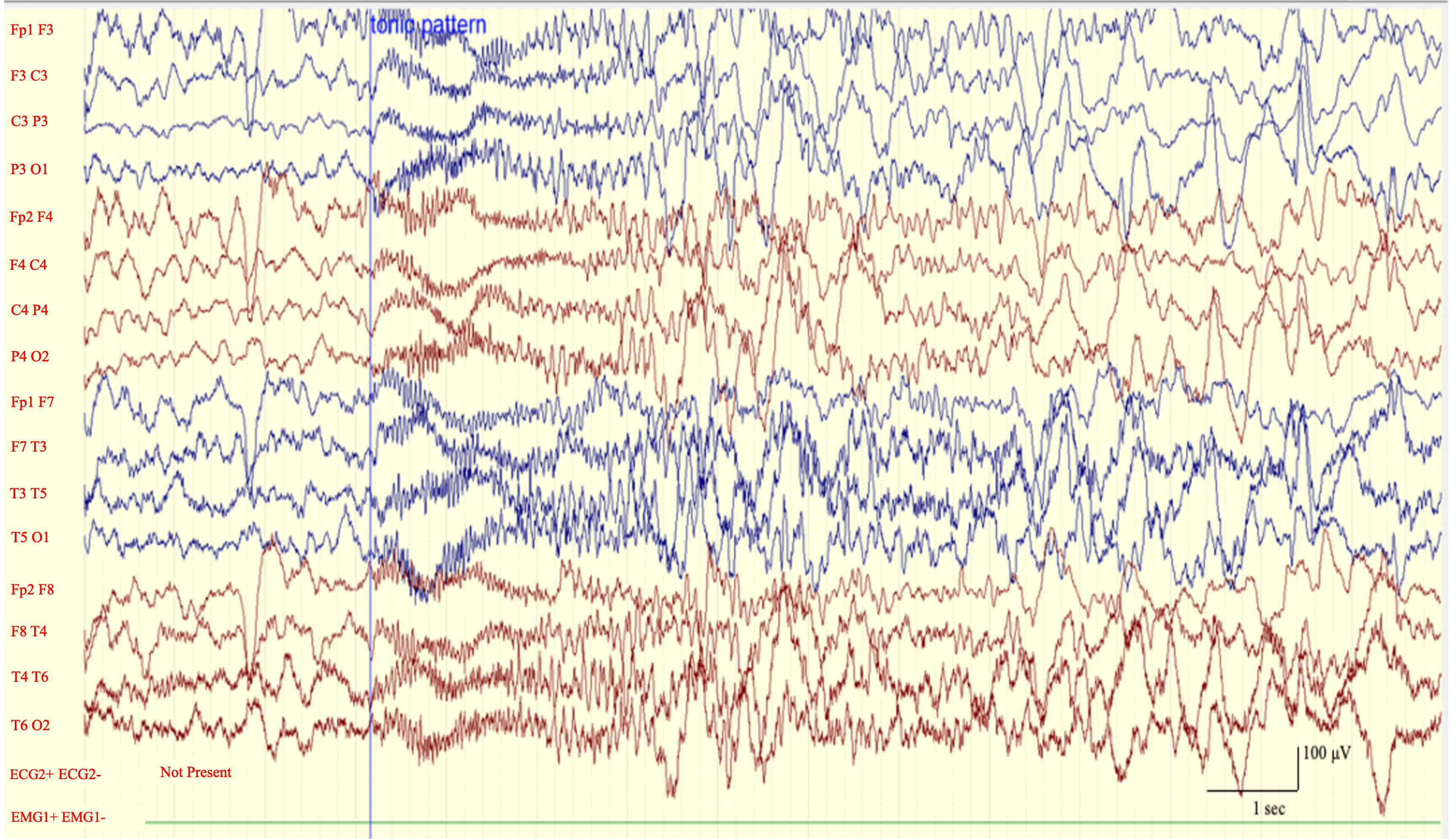
**Supplementary Figure 2.** Tonic pattern. Longitudinal bipolar montage, paper speed 15 mm/s, sensitivity 100 µV/cm (P4).


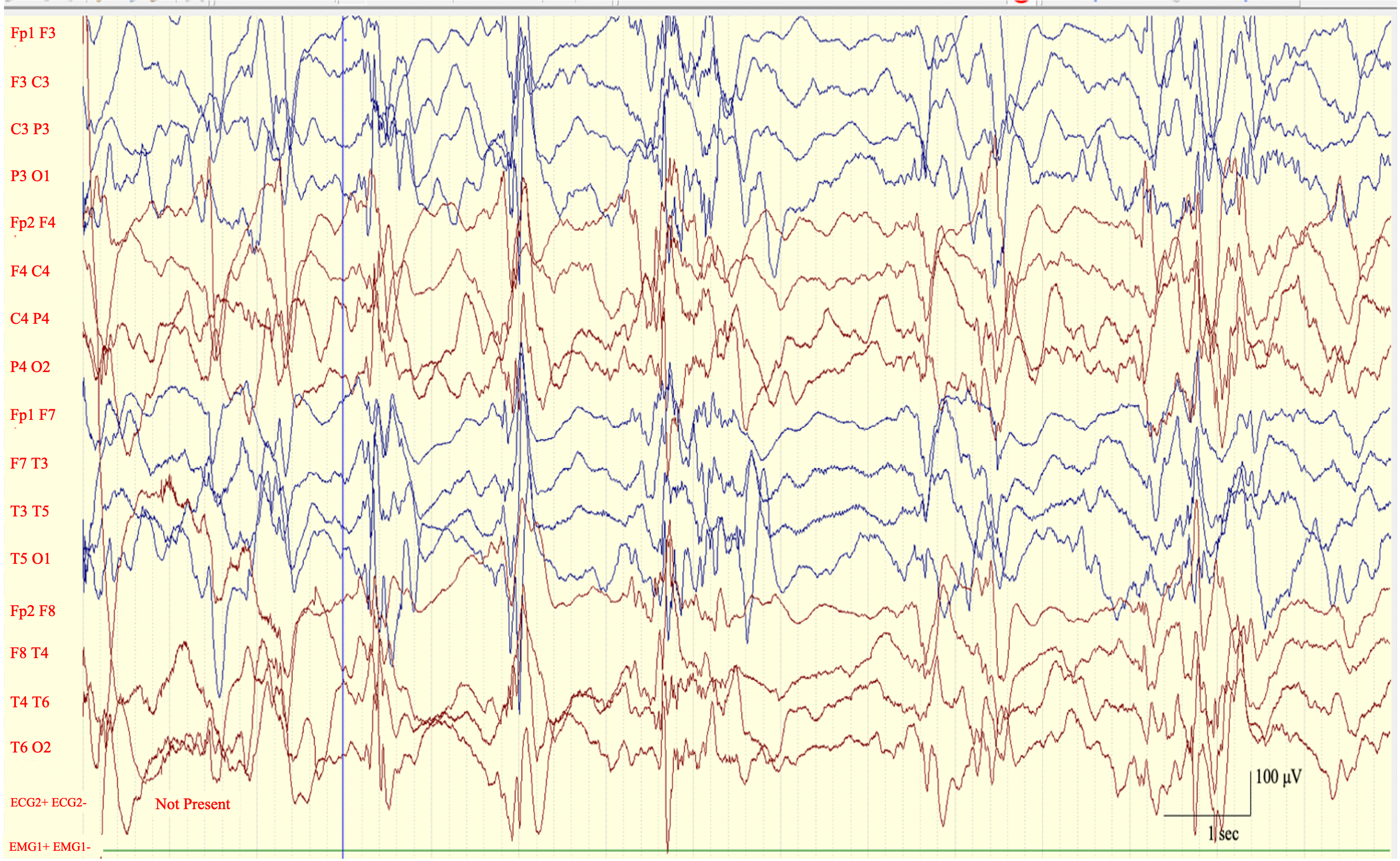


**Supplementary Figure 3.** Burst-supression pattern during sleep. Longitudinal bipolar montage, paper speed 15 mm/s, sensitivity 100 µV/cm (P4).
